# Creating a cancer genomics curriculum for pediatric hematology‐oncology fellows: A national needs assessment

**DOI:** 10.1002/cam4.3787

**Published:** 2021-02-23

**Authors:** Alise K. Murray, Rose B. McGee, Roya M. Mostafavi, Xiaoqing Wang, Zhaohua Lu, Jessica M. Valdez, Michael A. Terao, Kim E. Nichols

**Affiliations:** ^1^ Department of Oncology St. Jude Children's Research Hospital Memphis TN USA; ^2^ Department of Biostatistics St. Jude Children's Research Hospital Memphis TN USA; ^3^ Department of Pediatrics University of New Mexico Albuquerque NM USA; ^4^ Department of Pediatrics Division of Pediatric Hematology and Oncology Medstar Georgetown University Hospital Washington DC USA

**Keywords:** curriculum development, genomics, medical education, needs assessment, oncology

## Abstract

**Background:**

With the advent of next generation sequencing, tumor and germline genomic testing are increasingly being used in the management of pediatric cancer patients. Despite this increase in testing, many pediatric hematology–oncology (PHO) providers are not confident interpreting or utilizing tumor or germline genomic results to care for their patients.

**Methods:**

We developed and delivered a needs assessment survey to PHO program directors, attendings, and fellows in the United States to understand this deficiency, gather data on existing cancer genomics educational initiatives, and query preferences for creating a future curriculum.

**Results:**

The survey includes 31 (41%) of 74 invited PHO program directors, 110 (11%) of 1032 invited attendings, and 79 fellows. The majority of attending physicians and fellows responding to the survey agree that understanding tumor (95% attending physicians; 95% fellows) and germline (86% attending physicians; 94% fellows) genomic information is essential for their practice. However, only 9 of 31 (29%) responding programs report that they have an existing cancer genomics curriculum. Most program directors indicated that the ideal genomics curriculum would occur during the first year of fellowship and incorporate direct patient care, online modules, and problem‐based learning. Attending physicians and fellows identified that addressing indications for ordering tumor and germline genomic testing, counseling about the risks and benefits of such testing, and interpreting and individualizing clinical management based on tumor and germline results should be included in a future curriculum.

**Conclusion:**

The results of this study reveal a great need to develop a curriculum that can be offered across PHO fellowship programs to expand knowledge in the area of cancer genomics.

## Graphical abstract

A national needs assessment was performed to evaluate the perspectives of pediatric hematology‐oncology (PHO) fellowship program directors, attendings and fellows regarding development and deployment of a formal cancer genomics curriculum as a standard component of training. Only 29% of pediatric hematology‐oncology fellowship programs incorporate formal training in cancer genomics, yet most attending physicians and fellows report that tumor and germline genomic information is important for their clinical practice. The results of this study demonstrate the need to develop a cancer genomics curriculum for PHO fellows.

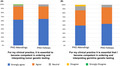

## 1 INTRODUCTION

As clinical next generation sequencing (NGS) of pediatric tumors has become less costly and more rapid, there has been an expansion in its use to streamline cancer diagnosis, refine prognosis, and guide treatment decisions.^1^, ^2^, ^3^ Indeed, NGS is increasingly being incorporated into the care of children with cancer, including newly diagnosed patients as well as those with relapsed/refractory disease.^1^ Despite this increase in use, in one recent study only 35% of pediatric hematology–oncology (PHO) providers felt confident interpreting, using, and discussing tumor NGS test results with families.^4^ In another study, only 62% of pediatric oncologists felt confident making treatment recommendations based on tumor NGS information.^5^

Germline NGS is also increasingly being offered to pediatric oncology patients to identify those with an underlying cancer predisposition. Germline results can guide cancer treatment decisions and provide crucial information about the risk to develop future cancers or non‐oncologic manifestations.^6^, ^7^, ^8^, ^9^ Recent large‐scale sequencing studies have revealed the presence of pathogenic or likely pathogenic germline variants associated with known cancer predisposition syndromes in at least 9–12% of children with cancer,^7^, ^8^, ^9^, ^10^ with this proportion likely to grow as new predisposition genes are identified.^7^, ^8^, ^9^, ^10^ Nevertheless, only 33% of PHO physicians at one institution were confident using germline genetic findings for patient care, and only 37% were comfortable discussing these results with patients and families.^5^

Although the Accreditation Council for Graduate Medical Education (ACGME) requires PHO fellows to receive “structured educational instruction in the related basic sciences,” which includes genetics,^11^ recent data show that PHO physicians lack confidence in applying genetic information to patient care.^4^, ^5^ This lack of confidence may be due to limited education about cancer genomics during fellowship training. As NGS becomes the standard‐of‐care, it is essential that PHO physicians are competent in understanding and using these results to care for their patients. Even so, there appears to be a significant knowledge gap among PHO providers.

To explore the perspectives of PHO physicians surrounding education in tumor and germline genomics, we completed a general and targeted needs assessment of PHO fellowship program directors, attending physicians, and fellows in the United States as part of the six steps of curriculum development.^6^ This assessment included identifying how PHO fellowship programs are currently carrying out cancer genomics education, as well as what they believe to be the ideal approach to such education. The needs identified through this study can be used to design a curriculum that will provide PHO fellows as well as faculty with the knowledge required to optimally incorporate tumor and germline genomic information into the care of their patients with cancer.^6^

## 2 METHODS

### 2.1 Participant recruitment

This study was approved by the Institutional Review Board at St. Jude Children's Research Hospital. An invitation to take part in this study was sent to potential participants via email. The invitation included a link to the corresponding online needs assessment survey.^12^ The first email invitation was sent on 28 April 2020, and a reminder invitation was sent 2 weeks later. The survey was open for 4 weeks. All emails were sent, and responses were tracked using Active Campaign.^13^

### 2.2 PHO fellowship program directors and fellows

The initial invitation to participate was sent to all 74 PHO fellowship training programs in the United States. For 70 of these programs, the invitation was sent directly to the program director for review and consideration of participation, as well as to the program coordinator so that it could be forwarded to the PHO fellows at their respective training programs. For four programs, the invitation was sent only to the program coordinator because contact information for the program director was not readily available. Email addresses were obtained from the ACGME accreditation data system^14^ or from the Fellowship and Residency Electronic Interactive Database (FREIDA).^15^ A link to the survey for fellows was also posted on the American Society of Pediatric Hematology Oncology online forum (ASPHO).

### 2.3 PHO attending physicians

The initial invitation email was also sent to 1103 PHO‐attending physicians. Seventy‐two emails (6.5%) were returned as undeliverable, four physicians sent an automatic reply with a new email address (0.3%), and three (0.3%) unsubscribed from the initial email. Accounting for these changes, the reminder invitation was sent to 1032 PHO‐attending physicians. Email addresses for attending physicians were extracted from the websites of PHO Divisions and Departments associated with each of the 74 fellowship programs. When PHO‐attending physician names, but not email addresses, were displayed on the websites, we predicted the email addresses according to their institutional email structure from other known email addresses for individuals at the institution, as found through various online sources.

### 2.4 Surveys

The surveys used in this study were developed by the study team, and the content was validated by seven clinical genomics experts (PHO physicians and genetic counselors) who were not associated with the project and from institutions other than St. Jude Children's Research Hospital. Content validity was established for 10 novel objective questions and 12 novel subjective questions.^16^, ^17^ Although only one question did not meet the previously defined validation criteria,^16^, ^17^ four objective questions and nine subjective questions were removed from the final survey to shorten the survey length. It was considered that the benefit gained from the answers to these 13 questions was not worth the risk of a reduced response rate. The PHO attending physicians and fellows survey included up to 20 questions, depending on how some of the questions were answered. One of these questions was optional. The PHO program director survey included up to 10 questions. Specifically, PHO fellowship directors at programs with an existing clinical genomics curriculum were asked 10 questions, 9 of which were mandatory, while PHO fellowship directors at programs without a clinical genomics curriculum were asked 5 mandatory questions. All of the surveys can be found in the Supplemental Materials.

### 2.5 Data analysis

Basic descriptive statistics were used to analyze survey results. Fisher's exact test was used to compare the answers to questions between the attendings and fellows. Bhapkar's test was used to compare the answers to questions about tumor and germline genomics. Two‐sided *P*‐values <0.05 were considered significant. All statistical analyses were conducted using R version 4.0.2.

## 3 RESULTS

### 3.1 Program directors

#### 3.1.1 Demographics and response rate

Thirty‐one of 74 possible PHO fellowship programs (42%) from 23 states and the District of Columbia completed the survey (Table S1).

Among these 31 programs, only 9 (29%) reported having existing cancer genomics curricula, ranging from 0 to 36 months in length. Each of the programs with a curriculum relied on local individuals with expertise in genetics and genomics such as geneticists, genetic counselors, oncologists, and pathologists to teach some, if not all, of the curriculum content. Eight program directors provided further information about the structure of their curricula, with all eight (100%) using in‐person lectures, five (63%) including direct patient care, and one (13%) incorporating problem‐based learning.

All program directors were asked to identify the characteristics that they considered ideal for a future cancer genomics curriculum for PHO fellows. Twenty‐two (71%) program directors indicated the first year of the fellowship training would be the best time to implement a cancer genomics curriculum. Several educational methods were elected as appropriate for inclusion, including direct patient care (25 program directors, 81%), online modules (24 program directors, 77%), problem‐based learning (20 program directors, 65%), recorded lectures viewed online (17 program directors, 55%), assigned readings (16 program directors, 52%), and a mobile phone application (5 program directors, 16%). In an optional free response question, several program directors mentioned the importance of flexibility in terms of scheduling lectures and other activities, and incorporating multifaceted learning.

### 3.2 PHO attendings and fellows

#### 3.2.1 Demographics and response rate

The survey was completed by 110 of 1032 of PHO attending physicians (11%). These attending physicians represented 41 institutions from 26 states and the District of Columbia (Table S1).

There are presumably 474 PHO fellows in the United States at 74 distinct programs based on fellowship match data from 2017 to 2019.^18^ Among these, 79 (17%) completed the survey. Based on how the survey was completed, it is not possible to determine how many fellows actually received the invitation to participate because the survey was anonymous and the program directors and coordinators did not notify the study team as to which PHO fellows they forwarded the survey. The 79 PHO fellows who participated in this study represent 31 PHO fellowship programs in 24 states and the District of Columbia (Table S1).

#### 3.2.2 Genomics curriculum relevance

**FIGURE 1:**
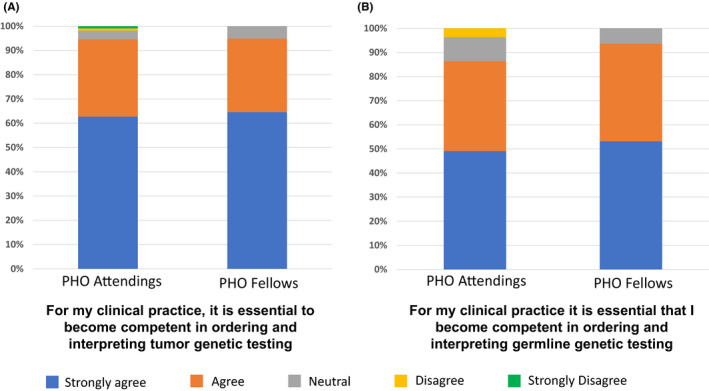
Relevance of cancer genomics to the practice of pediatric hematology oncology. PHO attendings and fellows were asked if they felt it was essential to their practice to become competent in ordering and interpreting tumor (A) and germline (B) genetic testing. The proportions of attendings and fellows responding to specific survey options are as shown.

PHO attendings and fellows report that genomics is very relevant to their clinical practice. Specifically, 104 of 110 (95%) attendings and 75 of 79 (95%) fellows strongly agreed or agreed that it was essential that they become competent in ordering and interpreting tumor genetic testing, and 95 of 110 (86%) attendings and 74 of 79 (94%) fellows strongly agreed or agreed that it was essential that they become competent in ordering and interpreting germline genetic testing (Figure 1). Two (2%) attending physicians did not agree that ordering and interpreting tumor genetic testing was relevant to their practice; one attending noted that there are already experts in genomics available to guide this testing at their institution and the other indicated that he/she does not often order or interpret clinical genomic testing for pediatric cancer patients. Similarly, four (4%) attending physicians who did not agree that ordering and interpreting germline genetic testing was relevant to their practice indicated that there is a geneticist/genetic counselor or other expert available to guide this testing at their institution.

#### 3.2.3 Local genomics expertise

Ninety‐eight (89%) attending physicians and 66 (84%) fellows indicated that they have an individual such as an oncologist or molecular pathologist at their institution to consult with prior to ordering tumor genetic testing. Similarly, 105 (95%) PHO attending physicians and 69 (87%) PHO fellows have access to a geneticist or genetic counselor with whom they can consult prior to ordering germline genetic testing.

#### 3.2.4 Genomics curriculum preferences

**FIGURE 2:**
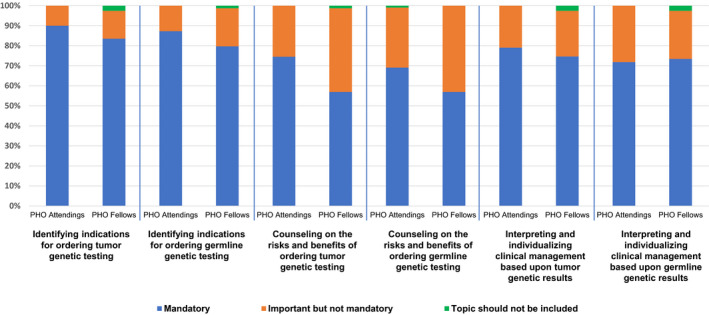
Importance of specific topics for inclusion in the ideal cancer genomics curriculum. PHO attendings and fellows were asked if various topics should be included in the ideal cancer genomics curriculum. The proportions of attendings and fellows responding to specific survey options are as shown.

When queried regarding the importance of specific topics for inclusion in a cancer genomics curriculum, the topics indicated as “mandatory” by attendings and fellows included: (i) identifying indications for ordering tumor genomic testing (90% of attendings and 84% fellows); (ii) identifying indications for ordering germline genomic testing (87% attendings and 80% fellows); (iii) interpreting and individualizing clinical management based upon tumor genomic results (79% attendings and 75% fellows); (iv) interpreting and individualizing clinical management based upon germline genomic results (72% attendings and 73% fellows); (v) counseling on the risks and benefits of tumor genomic testing (75% attendings and 57% fellows); and (vi) counseling on the risks and benefits of germline genomic testing (69% attendings and 57% fellows) (Figure 2 and Table S2). Notably, attendings from institutions where there is ready access to hereditary cancer expertise were significantly more likely to rate “Identifying indications for ordering germline testing” as a mandatory topic (71%) compared with attendings at institutions without such expertise (40%, *p* = 0.028).

In a free response question, several attending physicians indicated that ethical and financial considerations surrounding genetic testing should also be included in the curriculum as well as education on how to properly collect a family history.

## 4 DISCUSSION

Although tumor and germline genomic testing are becoming standard of care for pediatric cancer patients,^1^, ^2^, ^3^ many PHO providers are not certain about how to interpret or use the resulting information to care for their patients.^4^, ^5^ This deficit could be due to a lack of formal educational initiatives that focus on cancer genomic testing, analysis of genomic data, and the clinical and ethical implications of test results. It is essential that this deficit be addressed if providers are to effectively and ethically incorporate genomic data into the care of their pediatric cancer patients. To better understand this deficit, we queried PHO program directors, attendings, and fellows about their current educational initiatives in cancer genomics and explored the factors that they felt should be included in a future curriculum. Collectively, 104 of 110 (95%) responding attendings and 75 of 79 (95%) responding fellows report that understanding tumor genomic information is very relevant to their practice (Figure 1). Similarly, 95 of 110 (86%) attendings and 74 of 79 (94%) fellows felt the same way about understanding germline genomic information (Figure 1). Nevertheless, 22 of 31 (71%) responding programs do not yet have a formal cancer genomics curriculum for PHO fellows at their institution. Thus, there is a great need to develop such a curriculum that can be offered across PHO programs to expand knowledge in the area of cancer genomics.

Successful development of educational curricula in other fellowship programs has been achieved and may serve as a framework for development of a genomics‐focused curriculum for PHO fellows.^19^, ^20^ A needs assessment performed among pediatric emergency medicine fellowship directors identified the need for and led to development of a national patient safety curriculum for pediatric emergency medicine fellows.^19^ This curriculum was successful in increasing fellows' knowledge of patient safety issues and heightening awareness of safety issues and incorporation of safety strategies into clinical practice.^19^ Additionally, Reiss et al. reported incorporation of simulation‐based curricula regarding chemotherapy writing, bone marrow biopsy and intrathecal chemotherapy administration, and patient‐centered communication skills training in an adult Hematology–Oncology fellowship program.^20^ This study found that the workshop‐based curriculum led to increased knowledge and comfort of fellows with the elements of cancer care.^20^ Development and incorporation of a genomics curriculum for PHO fellows may similarly benefit trainees by increasing their knowledge, comfort, and utilization of genomic information in clinical practice.

It is interesting to note that attendings who have access to individuals with hereditary cancer expertise were significantly more likely to rate “Identifying indications for ordering germline testing” as a mandatory topic (70.5%) for inclusion in a cancer genomics curriculum compared with those without such access (40%, *p* = 0.028). This may indicate that these attendings recognize the value of this service and want to be involved in the process. By contrast, five attending physicians indicated that ordering and interpreting the results of tumor or germline testing were not relevant, as there already existed a genomics expert at their institution who was available to help guide and follow up on such testing. It is important to recognize that not all institutions will have access to these experts. Due to the rapid increase in genetic testing and limited number of genetic counseling training programs, there is currently a shortage of genetic counselors in the United States.^21^ While this shortage is projected to reach equilibrium between 2024 and 2030,^21^ PHO is not a primary specialty among genetic counselors.^22^ According to the National Society of Genetic Counselors 2020 Professional Status Survey, only 1% of genetic counselors work in pediatric oncology and 0.5% in adult and pediatric hematology.^22^ Therefore it may be challenging to recruit genetic counselors to work in the PHO setting. Based on this information, it is even more essential that PHO providers are educated in the area of germline genomics. Our data indicate that the majority of PHO physicians see value in gaining personal knowledge of tumor and germline genomics and better understanding how this information can be used to improve patient care.

In our survey, the majority of participants reported that identifying indications for ordering tumor and germline genetic testing, counseling on the risks and benefits of tumor and germline genetic testing, and interpreting and individualizing clinical management based on tumor and germline genetic results should be mandatory for inclusion in a future cancer genomics curriculum. Curiously, more attending physicians preferred mandatory inclusion (75%) of counseling on the risks and benefits of tumor genetic testing when compared with the fellows (57%, *p* = 0.014). Counseling is integral to obtaining consent for genetic testing of any kind^23^, ^24^, ^25^ and should address key topics such as the types of test results, the implications and management based on results, and the potential for and protections against genetic discrimination.^25^ The scope and limitations of the Genetic Information Nondiscrimination Act (GINA)^26^ must be thoroughly discussed when germline testing is being offered.^25^ GINA is a law that was enacted in 2008 that prevents certain health insurance companies and employers from discriminating against individuals who have genetic conditions.^25^ Additionally, ethical considerations including the familial implications of germline test results, discussion of testing minors for adult‐onset conditions, and potential for incidental findings such as consanguinity or non‐paternity should be reviewed prior to initiation of germline testing.^25^ Counseling regarding tumor genetic testing should include discussion of the potential to uncover variants that are likely to be of germline origin. This discrepancy between attending and fellow responses suggests that fellows require further training to fully appreciate the nuances and ethical implications of the tumor and germline genetic testing process.

Direct patient care (81%) was the most commonly selected option for inclusion in an ideal clinical genomics curriculum. Notably, of the 74 institutions currently offering a PHO fellowship, 46 also offer a Medical Genetics residency^15^ or a Genetic Counseling graduate program^27^ (Table S3). As medical genetics and genetic counselor training programs incorporate cancer predisposition education in their own curricula, they could serve as an important resource when developing a cancer genomics curriculum for PHO fellows. For example, PHO fellows could rotate along with medical genetics residents or genetic counseling students as they complete this component of their training.

With advancements in technology, creating a curriculum that is flexible, multifaceted, comprehensive, and available to every PHO fellowship program is possible. Today, distance and online learning are prevalent.^28^, ^29^ The incorporation of technology in the classroom has only increased with the COVID19 pandemic.^28^, ^29^, ^30^ Most PHO program directors indicate that it would be best to deliver a cancer genomics curriculum during the first year of fellowship, with online modules and direct patient care as primary components. An online module could include the other proposed learning models such as problem‐based learning, recorded lectures, and discussion of assigned readings. There are many existing resources (Table 1) that can be utilized as a part of, or in addition to, this curriculum. The use of distance learning and recorded lectures allows for students to learn from the most qualified individuals who may not be necessarily available to give in‐person lectures.^28^ Even at smaller institutions without local expertise in cancer genomics, patients could still receive consultation from experts in cancer genomics through the use of telehealth, as has been done in other specialties.^31^, ^32^, ^33^ Another point of access to expert knowledge in cancer genomics could be online information sharing platforms that allow subject matter experts to widely disseminate their expertise in a more rapid manner than through traditional peer‐reviewed publications.^34^ This type of platform has recently been used to disseminate expert knowledge surrounding how to care for oncology patients during the COVID19 pandemic.^34^

**TABLE 1:** Existing genomics resources.

Resource	Type	Content
ASCO Genetics Toolkit https://www.asco.org/practice‐policy/cancer‐care‐initiatives/genetics‐toolkit	CME courses & Tumor Boards	Background of Cancer Predisposition Syndromes; Testing Strategies, Risk Assessments, and Ethical Considerations
ASCP Training Residents in Genomics https://www.pathologylearning.org/trig/courses‐workshops	Working Group	General Genetics Education
National Human Genome Research Institute https://www.genome.gov/For‐Health‐Professionals/Provider‐Genomics‐Education‐Resources	Readings, Webinars, Curriculum	General Genomics Education
City of Hope Cancer Genomics Education Program https://www.cityofhope.org/education/health‐professional‐education/cancer‐genomics‐education‐program	Online Course	Hereditary Cancer Education
MIPOGG https://mipogg.com/	App	Cancer Predisposition Syndromes, When to refer to genetics
ASH Genomics resources https://www.hematology.org/education/educational‐programs/genom	Online Modules	Basic & disease‐specific hematology and oncology genomics

Despite its strengths, this study also has some limitations. First, we were not able to determine the true response rate among PHO fellows because we do not know how many fellows received the invitation to participate. To optimize the response rate, we kept the survey short and focused. As a result, only limited information was collected. Further, information could have been gathered regarding current strategies for how best to introduce PHO fellows to tumor and germline genomics. Similarly, it would have been interesting to know which programs had existing Developmental Therapeutics (DT) initiatives which rely heavily on generation and understanding of tumor genomic information. Accordingly, it is possible that PHO providers or fellows from institutions with DT initiatives might have answered questions differently than those from institutions without such initiatives. Last, only 10% of PHO attendings and 42% of PHO fellowship programs responded to this survey. Despite these incomplete response rates, these values are comparable to those observed in similar studies.^27^, ^28^, ^29^, ^30^, ^35^, ^36^ To the best of our knowledge, this effort, which used the first three steps of curriculum development,^6^ was the first to assess PHO program directors, attendings, and fellows for their interest in and needs for a cancer genomics curriculum.

## 5 CONCLUSION

This study reveals that the majority of PHO attendings and fellows recognize the importance of clinical cancer genomics and express a need for developing a curriculum to expand knowledge in the areas of tumor and germline testing, including how and when to order specific tests and how to interpret and act upon the results. Further efforts are needed to develop such a cancer genomics curriculum so that PHO providers can capitalize on the full potential of precision medicine to improve the diagnosis and management of children with cancer.

## CONFLICTS OF INTEREST

The authors have no conflicts of interest to disclose.

## AUTHOR CONTRIBUTIONS

Alise K. Murray performed conceptualization, data curation, formal analysis, investigation, methodology, project administration, validation, visualization, and writing original draft. Rose B. McGee performed conceptualization, data curation, methodology, validation, and writing – review and editing. Roya M. Mostafavi performed conceptualization, formal analysis, data curation, validation, and writing – review and editing. Xiaoqing Wang performed formal analysis, data curation, validation, and writing – review and editing. Zhaohua Lu performed formal analysis, data curation, validation, and writing – review and editing. Jessica M. Valdez performed conceptualization, cata curation, methodology, validation, and writing – review and editing. Michael A. Terao performed conceptualization, data curation, formal analysis, investigation, methodology, project administration, resources, validation, and writing – review and editing. Kim E. Nichols performed conceptualization, data curation, methodology, project administration, supervision, validation, and writing – review and editing.

## ETHICS

This study was approved by the Institutional Review Board at St. Jude Children's Research Hospital.

## Supporting information

Table S1

Table S2

Table S3

Supplementary Material

Supplementary Material

## Data Availability

The data that supports the findings of this study are available in the Supplementary Material of this article.
