# Supplementary material for: Creating a cancer genomics curriculum for pediatric hematology‐oncology fellows: A national needs assessment
Source: Cancer Med. 2021 Feb 23;10(6):2026–34. doi: 10.1002/cam4.3787 (PMC7957159; doi:10.1002/cam4.3787)
Supplement: Supplementary file 1 — Table S1 [file CAM4-10-2026-s004.docx]

***Supplemental Table 1.* Institutions, by Study Participants**

| **Program** | **State** | **Program Directors Participating** | **Attendings**  **Participating** | **Fellows**  **Participating** |
| --- | --- | --- | --- | --- |
| University of Alabama Medical Center (Birmingham)  Children’s Hospital of Alabama | AL | Yes | 3 | 2 |
| Phoenix Children’s Hospital | AZ | Yes | 1 | 0 |
| University of Arkansas for Medical Sciences  Arkansas Children’s Hospital | AR | No | 1 | 0 |
| Children’s Hospital of Los Angeles | CA | Yes | 1 | 4 |
| University of California Irvine  Children’s Hospital of Orange County | CA | Yes | 2 | 0 |
| Stanford University School of Medicine  Stanford Health Care  Lucille Packard | CA | Yes | 3 | 2 |
| University of California San Francisco  Benioff Children’s Hospital Oakland | CA | Yes | 0 | 2 |
| University of California San Diego  Rady Children’s Hospital | CA | No | 1 | 0 |
| University of Colorado  Children’s Hospital of Colorado | CO | Yes | 0 | 0 |
| Yale University School of Medicine  Yale-New Haven Medical Center | CT | Yes | 2 | 0 |
| University of Connecticut  Connecticut Children’s Medical Center | CT | No | 1 | 0 |
| George Washington University  Children’s National Medical Center | DC | No | 2 | 5 |
| Sidney Kimmel Medical College at Thomas Jefferson University  A. I. Dupont Hospital for Children | DE | Yes | 1 | 1 |
| University of Florida College of Medicine Jacksonville  Nemours | FL | Yes | 2 | 1 |
| University of Miami | FL | No | 1 | 0 |
| Emory University School of Medicine  Children’s Healthcare of Atlanta | GA | Yes | 3 | 6 |
| McGaw Medical Center of Northwestern University  Ann & Robert H. Lurie Children’s Hospital of Chicago | IL | No | 0 | 1 |
| University of Chicago | IL | Yes | 3 | 2 |
| Indiana University School of Medicine  Riley Hospital for Children | IN | No | 0 | 3 |
| University of Iowa Hospitals and Clinics | IA | Yes | 4 | 2 |
| University of Louisville School of Medicine | KY | No | 0 | 1 |
| Louisiana State University Health Sciences Center  Children’s Hospital | LA | No | 1 | 1 |
| National Capital Consortium  Walter Reed National Military Medical Center | MD | Yes | 1 | 0 |
| Johns Hopkins University School of Medicine  Bloomberg Children’s Center | MD | No | 0 | 4 |
| Boston Medical Center  Boston Children’s Hospital | MA | No | 9 | 0 |
| Michigan State University  Helen DeVos Children’s Hospital  Spectrum Health | MI | No | 1 | 0 |
| Children’s Hospital of Michigan | MI | Yes | 0 | 0 |
| University of Michigan Health System | MI | No | 3 | 0 |
| University of Minnesota  Masonic Children’s Hospital | MN | Yes | 2 | 3 |
| Mayo Clinic College of Medicine and Science | MN | No | 1 | 0 |
| Washington University School of Medicine  St. Louis Children’s Hospital Consortium | MO | Yes | 1 | 2 |
| Children’s Mercy Hospital | MO | No | 1 | 0 |
| University of Nebraska Medical Center College of Medicine | NE | No | 2 | 0 |
| University at Buffalo  John R. Oishei Children’s Hospital  Roswell Park Cancer Institute | NY | Yes | 0 | 0 |
| Albert Einstein College of Medicine  Children’s Hospital at Montefiore  Montefiore Medical Center | NY | Yes | 2 | 1 |
| University of Rochester | NY | No | 1 | 0 |
| Weill Cornell Medicine  New York Presbyterian Hospital  Memorial Sloan Kettering | NY | No | 1 | 0 |
| Columbia University Irving Medical Center  New York Presbyterian Hospital | NY | No | 0 | 4 |
| Duke University Hospital | NC | Yes | 4 | 3 |
| University of North Carolina Hospitals | NC | No | 1 | 0 |
| Cincinnati Children’s Hospital Medical Center | OH | Yes | 10 | 0 |
| Ohio State University  Nationwide Children’s Hospital | OH | Yes | 1 | 0 |
| University of Oklahoma College of Medicine | OK | Yes | 2 | 0 |
| Oregon Health & Science University | OR | Yes | 0 | 4 |
| University of Pittsburgh Medical Center  University of Pittsburgh Medical Center Children’s Hospital of Pittsburgh | PA | Yes | 2 | 3 |
| Pennsylvania State Milton S Hershey Medical Center  Penn State Children’s Hospital | PA | Yes | 0 | 0 |
| Brown University  Rhode Island Hospital  Hasbro Children’s Hospital | RI | Yes | 0 | 1 |
| Medical University of South Carolina | SC | No | 0 | 1 |
| University of Tennessee  St. Jude Children’s Research Hospital | TN | No | 16 | 3 |
| Vanderbilt University Medical Center | TN | No | 1 | 1 |
| University of Texas Southwestern Medical Center  Dallas Children’s | TX | Yes | 3 | 4 |
| Baylor College of Medicine  Texas Children’s Cancer and Hematology Centers  Texas Children’s Hospital | TX | Yes | 9 | 8 |
| Baylor College of Medicine San Antonio | TX | No | 1 | 0 |
| University of Utah Health  Primary Children’s Medical Center | UT | Yes | 3 | 1 |
| University of Virginia Medical Center | VA | Yes | 0 | 1 |
| University of Wisconsin Hospitals and Clinics | WI | No | 0 | 2 |

**Note**: The number under the Attendings and Fellows columns represents the number of attendings and fellows respectively from that institution that participated in this study.
