# Supplementary material for: Creating a cancer genomics curriculum for pediatric hematology‐oncology fellows: A national needs assessment
Source: Cancer Med. 2021 Feb 23;10(6):2026–34. doi: 10.1002/cam4.3787 (PMC7957159; doi:10.1002/cam4.3787)
Supplement: Supplementary file 2 — Table S2 [file CAM4-10-2026-s003.docx]

***Supplemental Table 2.* Importance of Topics for Inclusion in a Cancer Genomics Curriculum**

| **Please rate the importance of the following topics for possible inclusion in the curriculum for pediatric hematology/oncology fellows** | | | |
| --- | --- | --- | --- |
| Topic | Answer choices | PHO Attending Physicians N = 110 (%) | PHO Fellows N = 79 (%) |
| Identifying indications for ordering tumor genetic testing | Mandatory | 99 (90%) | 66 (83.54%) |
|  | Important but not mandatory | 11 (10%) | 11 (13.92%) |
|  | Topic should not be included | 0 (0%) | 2 (2.53%) |
| Identifying indications for ordering germline testing | Mandatory | 96 (87.27%) | 63 (79.75%) |
|  | Important but not mandatory | 14 (12.73%) | 15 (18.99%) |
|  | Topic should not be included | 0 (0%) | 1 (1.27%) |
| Counseling on the risks and benefits of tumor genetic testing | Mandatory | 82 (74.55%) | 45 (56.96%) |
|  | Important but not mandatory | 28 (25.45%) | 33 (41.77%) |
|  | Topic should not be included | 0 (0%) | 1 (1.27%) |
| Counseling on the risks and benefits of germline genetic testing | Mandatory | 76 (69.09% | 45 (56.96%) |
|  | Important but not mandatory | 33 (30.00%) | 34 (43.04%) |
|  | Topic should not be included | 1 (0.91%) | 0 (0%) |
| Interpreting and individualizing clinical management based upon tumor genetic results | Mandatory | 87 (79.09%) | 59 (74.68%) |
|  | Important but not mandatory | 23 (20.91%) | 18 (22.78%) |
|  | Topic should not be included | 0 (0%) | 2 (2.53%) |
| Interpreting and individualizing clinical management based upon germline genetic results | Mandatory | 79 (71.82%) | 58 (73.42% |
|  | Important but not mandatory | 31 (28.18%) | 19 (24.05%) |
|  | Topic should not be included | 0 (0%) | 2 (2.53%) |
