# Supplementary material for: Creating a cancer genomics curriculum for pediatric hematology‐oncology fellows: A national needs assessment
Source: Cancer Med. 2021 Feb 23;10(6):2026–34. doi: 10.1002/cam4.3787 (PMC7957159; doi:10.1002/cam4.3787)
Supplement: Supplementary file 3 — Table S3 [file CAM4-10-2026-s002.docx]

***Supplemental Table 3.* Institutions with a PHO Fellowship Program and a Genetics Residency or Genetic Counseling Graduate Program**

| **Program** | **State** | **Genetic Counseling Graduate Program** | **Medical Genetics Residency Program** |
| --- | --- | --- | --- |
| University of Alabama Medical Center (Birmingham)  Children’s Hospital of Alabama | AL | X | X |
| Phoenix Children’s Hospital | AZ |  |  |
| University of Arkansas for Medical Sciences  Arkansas Children’s Hospital | AR | X |  |
| Children’s Hospital of Los Angeles | CA |  |  |
| University of California Irvine  Children’s Hospital of Orange County | CA | X | X |
| Stanford University School of Medicine  Stanford Health Care  Lucille Packard | CA | X | X |
| University of California San Francisco  Benioff Children’s Hospital Oakland | CA | X | X |
| University of California San Diego  Rady Children’s Hospital | CA |  | X |
| UCLA David Geffen School of Medicine  UCLA Medical Center | CA | X | X |
| University of Colorado  Children’s Hospital of Colorado | CO | X | X |
| Yale University School of Medicine  Yale-New Haven Medical Center | CT |  | X |
| University of Connecticut  Connecticut Children’s Medical Center | CT |  | X |
| George Washington University  Children’s National Medical Center | DC |  | X |
| Sidney Kimmel Medical College at Thomas Jefferson University  A. I. Dupont Hospital for Children | DE | X |  |
| University of Florida College of Medicine Jacksonville  Nemours | FL |  |  |
| University of Florida College of Medicine Gainesville | FL |  |  |
| University of Miami  Miller School of Medicine  Jackson Health System | FL |  | X |
| Emory University School of Medicine  Children’s Healthcare of Atlanta | GA | X | X |
| McGaw Medical Center of Northwestern University  Ann & Robert H. Lurie Children’s Hospital of Chicago | IL | X |  |
| University of Chicago | IL |  |  |
| University of Illinois | IL |  |  |
| Indiana University School of Medicine  Riley Hospital for Children | IN | X | X |
| University of Iowa Hospitals and Clinics | IA |  | X |
| University of Louisville School of Medicine | KY |  |  |
| Louisiana State University Health Sciences Center  Children’s Hospital | LA |  |  |
| National Capital Consortium  Walter Reed National Military Medical Center | MD |  |  |
| Johns Hopkins University School of Medicine  Bloomberg Children’s Center | MD | X | X |
| Boston Medical Center  Boston Children’s Hospital | MA | X |  |
| Michigan State University  Helen DeVos Children’s Hospital  Spectrum Health | MI |  |  |
| Children’s Hospital of Michigan | MI |  |  |
| University of Michigan Health System | MI | X | X |
| University of Minnesota  Masonic Children’s Hospital | MN | X |  |
| Mayo Clinic College of Medicine and Science | MN |  | X |
| University of Mississippi Medical Center | MS |  | X |
| Washington University School of Medicine  St. Louis Children’s Hospital Consortium | MO | X | X |
| St. Louis University School of Medicine | MO |  |  |
| Children’s Mercy Hospital | MO |  |  |
| University of Nebraska Medical Center College of Medicine | NE | X | X |
| Hackensack University Medical Center | NJ |  |  |
| University at Buffalo  John R. Oishei Children’s Hospital  Roswell Park Cancer Institute | NY |  |  |
| Albert Einstein College of Medicine  Children’s Hospital at Montefiore  Montefiore Medical Center | NY |  | X |
| University of Rochester | NY |  |  |
| Weill Cornell Medicine  New York Presbyterian Hospital  Memorial Sloan Kettering | NY |  | X |
| Columbia University Irving Medical Center  New York Presbyterian Hospital | NY | X | X |
| New York University Grossman School of Medicine  New York University Langone Medical Center | NY |  |  |
| New York Medical College  Westchester Medical Center | NY |  |  |
| Zucker School of Medicine at Hofstra  Northwell at Cohen Children’s Medical Center  Cohen Children’s Medical Center of New York | NY |  |  |
| Duke University Hospital | NC |  | X |
| University of North Carolina Hospitals | NC | X | X |
| Cincinnati Children’s Hospital Medical Center | OH | X | X |
| Ohio State University  Nationwide Children’s Hospital | OH | X | X |
| Northeast Ohio Medical University  Children’s Hospital Medical Center of Akron | OH |  |  |
| Case Western Reserve University  University Hospitals Cleveland Medical Cneter  Rainbow Babies and Children’s Hospital | OH | X | X |
| Cleveland Clinic Foundation | OH |  |  |
| University of Oklahoma College of Medicine | OK | X | X |
| Oregon Health & Science University | OR |  | X |
| University of Pittsburgh Medical Center  University of Pittsburgh Medical Center Children’s Hospital of Pittsburgh | PA | X | X |
| Pennsylvania State Milton S Hershey Medical Center  Penn State Children’s Hospital | PA |  |  |
| Children’s Hospital of Philadelphia  University of Pennsylvania | PA | X | X |
| Brown University  Rhode Island Hospital  Hasbro Children’s Hospital | RI |  |  |
| Medical University of South Carolina | SC |  |  |
| University of Tennessee  St. Jude Children’s Research Hospital | TN |  |  |
| Vanderbilt University Medical Center | TN | X | X |
| University of Texas Southwestern Medical Center  Dallas Children’s | TX |  | X |
| Baylor College of Medicine  Texas Children’s Cancer and Hematology Centers  Texas Children’s Hospital | TX | X | X |
| Baylor College of Medicine San Antonio | TX |  |  |
| University of Texas Health Science Center at Houston  MD Anderson Cancer Center | TX | X | X |
| University of Texas Health Science Center San Antonio Joe and Teresa Lozano Long School of Medicine  University of Texas Health Science Center | TX |  |  |
| University of Utah Health  Primary Children’s Medical Center | UT | X | X |
| University of Virginia Medical Center | VA |  |  |
| Virginia Commonwealth University Health System | VA | X | X |
| University of Washington  Seattle Children’s Hospital | WA | X | X |
| University of Wisconsin Hospitals and Clinics | WI | X | X |
| Medical College of Wisconsin Affiliated Hospitals | WI | X | X |
