# Supplementary material for: Creating a cancer genomics curriculum for pediatric hematology‐oncology fellows: A national needs assessment
Source: Cancer Med. 2021 Feb 23;10(6):2026–34. doi: 10.1002/cam4.3787 (PMC7957159; doi:10.1002/cam4.3787)
Supplement: Supplementary file 4 — Supplementary Material [file CAM4-10-2026-s001.pdf]

We are creating a curriculum to teach pediatric hematology/oncology fellows about tumor and germline genomics.

Your answers to the questions below will help us to determine what we will include in the curriculum.

Once the curriculum is created, we will be freely sharing it with all pediatric hematology/oncology fellowship programs.

* 1. Please enter the name of your institution

* 2. Is there an individual with cancer genetics expertise at your institution (such as an oncologist or molecular pathologist) to consult with prior to or after ordering **tumor** genetic testing?

- Yes
- No

* 3. Is there an individual with hereditary cancer expertise at your institution (such as a geneticist, genetic counselor) to consult with prior to or after ordering **germline** genetic testing?

- Yes
- No

* 4. Please rate the importance of the following topics for possible inclusion in the curriculum for pediatric hematology/oncology fellows

	Mandatory	Important but not mandatory	Topic should not be included
Identifying indications for ordering **tumor** genetic testing	◯	◯	◯
Identifying indications for ordering **germline** testing	◯	◯	◯
Counseling on the risks and benefits of **tumor** genetic testing	◯	◯	◯
Counseling on the risks and benefits of **germline** genetic testing	◯	◯	◯
Interpreting and individualizing clinical management based upon **tumor** genetic results	◯	◯	◯
Interpreting and individualizing clinical management based upon **germline** genetic results	◯	◯	◯

5. Please list any other topics that should be included in the curriculum

* 6. A 4 year old boy is recently diagnosed with hepatoblastoma. While taking a history you learn that his parents struggled with infertility and utilized *in vitro fertilization* (IVF) to conceive. Upon physical examination, you notice that your patient's right leg is noticeably larger than his left.

What genetic condition is at the top of your differential?

- ◯ Beckwith Wiedemann Syndrome
- ◯ Gorlin syndrome
- ◯ Familial Adenomatous Polyposis
- ◯ Li Fraumeni syndrome

* 7. You decide to order **germline** genetic testing for the patient in the previous question. Which test is most likely to confirm the diagnosis?

  - DNA Methylation analysis
  - Single gene sequencing
  - Whole exome sequencing
  - Chromosome microarray (SNP, aCGH)

* 8. Given the most likely genetic diagnosis, what other tumor is this patient at risk to develop?

  - Wilms tumor
  - Neurofibromas
  - Thyroid cancer
  - Basal cell carcinoma

* 9. An 18 year old boy is diagnosed with a malignant peripheral nerve sheath tumor. **Tumor** testing results identify an NF1 mutation. What test or procedure can be performed to determine whether the patient has neurofibromatosis?

  - Physical exam
  - Whole body MRI
  - Audiology evaluation
  - Chromosome microarray (SNP, aCGH) on the blood

* 10. The family of a 12 year old African American girl with recently relapsed B-cell acute lymphoblastic leukemia elects to proceed with **tumor** testing of a leukemic bone marrow sample. The testing detects a *TP53* mutation at a 47% allele frequency. What does allele frequency refer to?

  - Out of 100 total sequence reads, 47 of them had the mutation
  - There is a 47% chance that the mutation is **germline** in origin
  - This mutation is seen in 47% of B-ALL cases
  - This mutation is seen among 47% of African American individuals

* 11. The parents of the child in question 10 wish to know whether the *TP53* mutation is **germline** in origin. Which of the following is the most appropriate tissue to test to address this question?

  - Skin fibroblasts
  - Saliva
  - Peripheral blood
  - Buccal cells

* 12. Please select the answer that best reflects your agreement or disagreement with the statement below:

"For my clinical practice, it is essential to become competent in ordering and interpreting **tumor** genetic testing."

Strongly agree	Agree	Neutral	Disagree	Strongly Disagree
○	○	○	○	○

Other (please specify)

* 13. Why do you believe it is not essential to become competent at ordering and interpreting **tumor** genetic testing?

- ◯ An oncologist or molecular pathologist or other expert in genomics will often be available to guide this testing
- ◯ I do not often order or interpret clinical genomic information in pediatric cancer patients
- ◯ I've not found clinical genomic information to change the way I manage pediatric cancer patients

Other (please specify)

* 14. Please select the answer that best reflects your agreement or disagreement with the statement below:

"For my clinical practice, it is essential to become competent at ordering and interpreting **germline** genetic testing."

Strongly agree	Agree	Neutral	Disagree	Strongly Disagree
○	○	○	○	○

* 15. Why do you believe it is not essential to become competent at ordering and interpreting **germline** genetic testing?

- ○ A geneticist/genetic counselor or other expert in genomics will often be available to guide this testing
- ○ I do not often order or interpret clinical genomic information in pediatric cancer patients
- ○ I've not found clinical genomic information to change the way I manage pediatric cancer patients

Other (please specify)

