# Supplementary material for: Creating a cancer genomics curriculum for pediatric hematology‐oncology fellows: A national needs assessment
Source: Cancer Med. 2021 Feb 23;10(6):2026–34. doi: 10.1002/cam4.3787 (PMC7957159; doi:10.1002/cam4.3787)
Supplement: Supplementary file 5 — Supplementary Material [file CAM4-10-2026-s005.pdf]

We are creating a curriculum to teach pediatric hematology/oncology fellows about tumor and germline genomics.

Your answers to the questions below will help us to determine what we will include in the curriculum.

Once the curriculum is created, we will be freely sharing it with all pediatric hematology/oncology fellowship programs.

* 1. Please enter the name of your institution

[ ]

* 2. During which year of fellowship would it be best to implement a cancer genomics curriculum?

- ○ 1st year
- ○ 2nd year
- ○ 3rd year

* 3. Is there access to personnel with cancer genomics expertise who could teach this curriculum (such as a geneticist, genetic counselor, oncologist, or molecular pathologist)?

- ○ Yes
- ○ No

* 4. Please select the learning methods from the list below that you think would be most effective for this planned curriculum (you can select as many choices as you want):

- ☐ Online Modules (Webinars): Interactive online sessions with a speaker
- ☐ Recorded Lectures viewed online
- ☐ Patient Care: Taking care of actual patients with cancer predisposition syndromes
- ☐ Assigned Readings
- ☐ Problem Based Learning: Small groups are given a case description and learn about various topics elicited by the case
- ☐ Mobile phone application

Other (please specify) or explain why you chose the method(s) above:

[ ]

* 5. Is there currently a curriculum to teach cancer genomics to your fellows?

- ◯ Yes
- ◯ No

* 6. How long do the fellows participate in your clinical genomics curriculum?

- ◯ 0-6 months
- ◯ 7-12 months
- ◯ 13-18 months
- ◯ 19-24 months
- ◯ 25-30 months
- ◯ 31-36 months

* 7. Please check all of the learning methods that are a part of your curriculum:

- ☐ Online Modules (Webinars)
- ☐ Lectures viewed online
- ☐ In person lectures
- ☐ Patient Care
- ☐ Assigned Readings
- ☐ Problem Based Learning: Small groups are given a case description and learn about various topics elicited by the case
- ☐ Mobile phone application

Other (please specify)

* 8. Do personnel with cancer genomics expertise (i.e. geneticists, genetic counselors, oncologist with this expertise, pathologist with this expertise) help teach this curriculum?

- ◯ Yes
- ◯ No

* 9. What percentage of the curriculum is taught by personnel with cancer genomics expertise (i.e. geneticists, genetic counselors)?

- ◯ 0-20%
- ◯ 21-40%
- ◯ 41-60%
- ◯ 61-80%
- ◯ 81-100%

10. Please describe any other aspects of your curriculum that has not been covered in the above questions
